# Reproducibility of the external surface position in left‐breast DIBH radiotherapy with spirometer‐based monitoring

**DOI:** 10.1120/jacmp.v15i1.4494

**Published:** 2014-01-04

**Authors:** Aurora Fassi, Giovanni B. Ivaldi, Ilaria Meaglia, Patrizia Porcu, Paola Tabarelli de Fatis, Marco Liotta, Marco Riboldi, Guido Baroni

**Affiliations:** ^1^ Dipartimento di Elettronica Informazione e Bioingegneria, Politecnico di Milano Milano Italy; ^2^ Department of Radiation Oncology Fondazione Salvatore Maugeri Pavia Italy; ^3^ Medical Physics Fondazione Salvatore Maugeri Pavia Italy; ^4^ Bioengineering Unit CNAO Foundation Pavia Italy

**Keywords:** left‐breast cancer, DIBH radiotherapy, spirometric monitoring, optical tracking, surface reproducibility

## Abstract

Deep inspiration breath hold (DIBH) in left‐sided breast cancer radiotherapy treatments allows for a reduction in cardiac and pulmonary doses without compromising target coverage. The selection of the most appropriate technology for DIBH monitoring is a crucial issue. We evaluated the stability and reproducibility of DIBHs controlled by a spirometric device, by assessing the variability of the external surface position within a single DIBH (intra‐DIBH) and between DIBHs performed in the same treatment session (intrafraction) or in different sessions (interfraction). The study included seven left‐breast cancer patients treated with spirometer‐based DIBH radiotherapy. Infrared optical tracking was used to record the 3D coordinates of seven to eleven passive markers placed on the patient's thoraco‐abdominal surface during 29‐43 DIBHs performed in six to eight treatment sessions. The obtained results showed displacements of the external surface between different sessions up to 6.3 mm along a single direction, even at constant inspired volumes. The median value of the interfraction variability in the position of breast passive markers was 2.9 mm (range 1.9‐4.8 mm) in the latero‐lateral direction, 3.6 mm (range 2.2‐4.6 mm) in the antero‐posterior direction, and 4.3 mm (range 2.8‐6.2 mm) in the cranio‐caudal direction. There were no significant dose distribution variations for target and organs at risk with respect to the treatment plan, confirming the adequacy of the applied clinical margins (15 mm) to compensate for the measured setup uncertainties. This study demonstrates that spirometer‐based control does not guarantee a stable and reproducible position of the external surface in left‐breast DIBH radiotherapy, suggesting the need for more robust DIBH monitoring techniques when reduced margins and setup uncertainties are required for improving normal tissue sparing and decreasing cardiac and pulmonary toxicity.

PACS number: 87.55.Km

## I. INTRODUCTION

Postoperative radiotherapy following breast conserving surgery significantly reduces the risk of local recurrences and improves long‐term survival.^(1)^ However, radiation‐induced cardiac and pulmonary complications represent a primary concern, particularly for left‐sided breast cancer patients.^(2)^ A clinically applicable protocol in left‐breast radiotherapy is the deep inspiration breath hold (DIBH), which allows increasing the distance between heart and breast and reducing lung density.^3^, ^4^ Dosimetric studies in left‐sided breast cancer patients treated with DIBH techniques showed a reduction of cardiopulmonary doses (up to 2.5 Gy) with respect to conventional free‐breathing treatments, without compromising target coverage.^5^, ^6^ Due to the radiobiological requirement of dose fractionation and to the need of multiple DIBHs for dose delivery in each therapy session, intra‐ and interfraction repeatability of DIBHs with respect to the treatment plan is a crucial issue, if inadequate clinical margins are applied. The selection of the most appropriate technology for DIBH monitoring is, therefore, essential to ensure the effectiveness of radiation therapy.

Different methods have been proposed to monitor the stability and reproducibility of repeated DIBHs during radiotherapy treatments. The most common technique uses spirometric devices that measure the volume of inspired and expired air through differential pressure transducers.^(7)^ DIBH can also be monitored by using external respiratory surrogates that capture the motion of the patient's thoraco‐abdominal surface. For example, infrared (IR) optical localizers can record the trajectory of single or multiple passive markers placed on the patient's skin near the irradiated target.^8^, ^9^, ^10^ A third technique involves the use of laser‐ or video‐based surface imaging systems, which can provide detailed breathing‐related information by dynamically scanning the entire patient's surface.^11^, ^12^, ^13^, ^14^ Three‐dimensional surface imaging has also been recently applied to quantify setup uncertainties in left‐breast DIBH radiotherapy,^(15)^ as external breast surface can be considered a reliable surrogate for the superficial mammary gland.^(16)^

The aim of this study was to evaluate DIBH stability and reproducibility during left‐breast radiation treatments under the control of a commercial spirometric device. DIBH performance analysis was based on noninvasive IR optical tracking of the external breast surface, by measuring the variability in the 3D position of multiple passive markers during repeated DIBHs at tolerated inspired volumes. The ability of spirometer‐based monitoring to ensure a stable and reproducible patient setup was assessed by evaluating the variability of passive marker positions within a single DIBH (intra‐DIBH stability) and between DIBHs performed in the same treatment session (intrafraction reproducibility) or in different sessions (interfraction reproducibility). In addition, we investigated the differences in the DIBH variability of thoracic and abdominal surface regions under spirometric guidance. The dosimetric consequences within target and organs at risk (OARs) associated with the measured DIBH uncertainties were also evaluated.

## II. MATERIALS AND METHODS

### A. Clinical protocol

**Figure 1:**
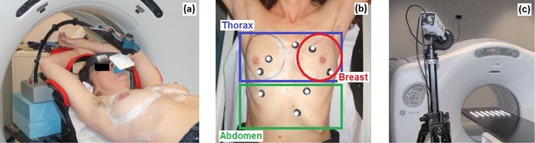
SpiroDyn'RX system (a) connected to the patient through a mouthpiece, equipped with video‐glasses for visual guidance. Multiple radiopaque/passive markers (b) placed on the patient's surface, grouped in left‐breast, thoracic, and abdominal regions. IR camera (c) of the optoelectronic localizer and grid phantom used for camera calibration.

Seven left‐sided breast cancer patients undergoing whole‐breast tangential radiotherapy with DIBH technique were selected for the study. A dedicated spirometric device (SpiroDyn'RX, Dyn'R, Muret Cedex, France), shown in Fig. 1(a), was used for DIBH control based on the measured volume of inspired air.^(17)^ A spirometry training session was performed before the CT simulation, to evaluate the patients’ ability to repeatedly hold their breath at constant forced inspiration for at least 20 seconds. For each patient, the DIBH reference volume was set to 90% of their maximum inspiratory capacity reached during training, as indicated for the SpiroDyn'RX system. The gating window was centered in the selected volumetric level, with a tolerance interval of ±0.1 liters. Visual feedback was provided to the patients with the aid of videoglasses (Fig. 1(a)), which displayed the breathing pattern and the selected DIBH gating window.

To quantitatively assess the reproducibility of the external surface position during planning and treatment DIBHs, multiple skin landmarks (nevi or scars) were selected on the patients’ thoraco‐abdominal surface. The number of landmarks per patient varied from seven to eleven, depending on the number of clearly visible nevi or scars that could be identified on the patients’ skin. For each patient, at least one landmark was located on the target left‐breast. Radiopaque markers (BTS Bioengineering, Milano, Italy) containing a metallic material were placed on the identified landmarks during CT simulation (Fig 1(b)). A photo of the marked patients’ surface was taken at the time of planning, in order to allow the recognition of the corresponding skin landmarks during the following treatment sessions.

All patients underwent two consecutive helical CT scans, each with 2.5 mm slice thickness. The first CT acquisition was performed in free‐breathing, and the second one within a single DIBH. The free‐breathing CT dataset was used to set the isocenter position for the daily patient setup, while the breath hold CT image was used for treatment planning (Philips Pinnacle v9.0, Philips Radiation Oncology Systems, Fitchburg, WI). The clinical target volume (CTV) was defined as the whole breast, and the planning target volume (PTV) was defined as the CTV plus a 15 mm margin to account for patient setup errors, breast deformation, and beam lateral penumbra. The CTV‐PTV margin was manually reduced near the heart and lungs to meet the following dose constraints, based on the National Surgical Adjuvant Breast and Bowel Project (NSABP) B‐39 / Radiation Therapy Oncology Group (RTOG) 0413 protocol:^(18)^

- dose received by 95% of the $\text{CTV}\;(D_{95\%})$ should be greater than 95% of the prescription dose;
- volume of heart receiving $5\;\text{Gy}\;(V_{5\text{Gy}})$ should be less than 5%;
- dose received by 1 cm^3^ of contralateral breast $\left(D_{1\text{cc}}\right)$ should be less than 7% of the prescription dose; and
- volume of ipsilateral lung receiving $20\;\text{Gy}\;(V_{20\text{Gy}})$ should be less than 20%.

At the beginning of each treatment session, passive markers with an IR‐reflective coating (BTS Bioengineering, Milano, Italy) were positioned on the corresponding skin landmarks selected at the time of planning (Fig. 1(b)). For patient setup, an initial laser‐based alignment was performed on skin tattoos in free‐breathing. Patient positioning was verified during DIBHs by checking the source‐skin distance (SSD) and by acquiring MV electronic portal images at treatment angles. Positioning errors were corrected by applying the appropriate shifts to the treatment couch (1 mm spatial resolution). The whole breast was treated with 2 to 4 opposed tangential photon beams at 2.25 Gy/fraction, for a total of 45 Gy delivered in 20 fractions over four weeks. Beam energies of 6 MV were used, with the addition of 15 MV beams in specific cases. High‐dose rates of 500‐600 MU/minute were applied to limit the delivery time up to 20 seconds per beam.

During the whole treatment session, the trajectories of passive markers placed on the patients’ surface were acquired by means of an IR optoelectronic localizer (ELITE, BTS Bioengineering, Milano, Italy), used in a dual camera configuration with a sample rate of 50 Hz (Fig. 1(c)). The 3D reconstruction of passive marker coordinates required the calibration of the stereocamera parameters, obtained with the Direct Linear Transformation method.^(19)^ The daily calibration procedure consisted of acquiring a $36\times 36\;{\text{cm}}^{2}$ grid phantom with 7×7 equispaced passive markers (Fig 1(c)). During calibration, the phantom was positioned on the treatment couch, aligning the central passive marker with the isocentric laser lines and shifting the couch at five different heights (with 5 cm step). The reference system axes of the calibrated optoelectronic localizer coincided with the latero‐lateral (LL), antero‐posterior (AP), and cranio‐caudal (CC) directions of the linac unit.

### B. DIBH performance analysis

**Figure 2:**
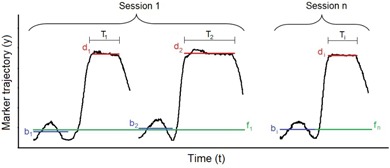
Exemplifying trajectory of a passive marker along a spatial direction. The parameters used for the estimation of the DIBH variability indices are represented in the Figure.

For practical reasons, only six to eight out of 20 treatment sessions were monitored for each patient. Each monitored session included at least four DIBHs: once during the SSD check, once during the portal image acquisition, and once per tangent field during treatment. The 3D coordinates of passive markers were continuously acquired by the IR optoelectronic localizer for the duration of DIBHs, including at least one free‐breathing cycle preceding each DIBH. The stability and reproducibility of the external surface position during repeated DIBHs was quantitatively evaluated by computing the following set of variability indices, illustrated in Fig. 2:

i) Intra‐DIBH stability, estimated as the 5th‐95th percentile range of the passive marker coordinates acquired within a single DIBH. The intra‐DIBH stability $S_{i}^{m}$ of the m‐th passive marker for the i‐th DIBH, performed in the temporal window $T_{i}$, was computed as:

(1) $$S_{i}^{m}=95_{t\in T_{i}}^{th}\left\{y{\left(t\right)}^{m}\right\}-5_{t\in T_{i}}^{th}\left\{y{\left(t\right)}^{m}\right\}$$

where $y({t)}^{m}$ represents the marker coordinate along a spatial direction.

ii) Intrafraction reproducibility, estimated as the 5th‐95th percentile range of the passive marker intra‐DIBH positions associated with all DIBHs occurred within the same session. The intrafraction reproducibility $V_{n}^{m}$ of the m‐th passive marker in the n‐th session $S_{n}$ was obtained with the following equation:

(2) $$V_{n}^{m}=95_{i\in S_{n}}^{th}\left\{d_{i}^{m}\right\}-5_{i\in S_{n}}^{th}\left\{d_{i}^{m}\right\}$$

where $d_{i}^{m}$ is the intra‐DIBH position of the m‐th passive marker for the i‐th DIBH, computed as the median value of the passive marker coordinates acquired within the temporal window $T_{i}$:

(3) $$d_{i}^{m}=\underset{t\in T_{i}}{median}\left\{y{\left(t\right)}^{m}\right\}$$

For the DIBHs performed during SSD control and portal imaging, the intra‐DIBH positions were corrected for the applied setup shifts, to allow the comparison with postsetup DIBHs.

iii) Interfraction reproducibility, estimated as the 5th‐95th percentile range of the passive marker intra‐DIBH positions associated with all DIBHs performed in all sessions. The interfraction reproducibility $W^{m}$ of the m‐th passive marker was defined as:

(4) $$W^{m}=95_{i\in S_{n}}^{th}\left\{d_{i}^{m}-f_{n}^{m}\right\}-5_{i\in S_{n}}^{th}\left\{d_{i}^{m}-f_{n}^{m}\right\},\;n=[1,N]$$

where *N* is the total number of monitored treatment sessions. The term $f_{n}^{m}$ is the free‐breathing baseline of the m‐th passive marker for the n‐th session, which was subtracted from the intra‐DIBH positions in order to compensate for possible inaccuracies in marker placement and for residual setup errors. The baseline $f_{n}^{m}$ was obtained by mediating the passive marker free‐breathing positions $b_{i}^{m}$ associated with each DIBH performed in the n‐th session:

(5) $$f_{n}^{m}=\underset{t\in S_{n}}{median}\left\{b_{i}^{m}\right\}$$

The free‐breathing position $b_{i}^{m}$ for the i‐th DIBH was computed as the median value of the passive marker coordinates acquired within all monitored free‐breathing cycles that precede the DIBH. In order to validate the use of passive marker free‐breathing positions as baseline, the free‐breathing stability $F^{m}$ of each m‐th passive marker was assessed by computing the median distance between the baseline $f_{n}^{m}$ and the free‐breathing positions $b_{i}^{m}$ associated with all i‐th DIBHs performed in the n‐th session:

(6) $$F^{m}=\underset{t\in S_{n}}{median}\left\{f_{n}^{m}-b_{i}^{m}\right\},\;n=[1,N]$$

For each patient, the described variability indices were mediated over all passive markers. To investigate the differences in the breathing patterns of thoracic and abdominal surface compartments, the variability indices were also separately estimated by grouping passive markers placed on patients’ left‐breast, thorax, and abdomen. The line connecting the right and left submammary sulcus was used to distinguish thoracic and abdominal markers, as depicted in Fig. 1(b).

Dosimetric consequences caused by the measured surface uncertainties were evaluated following the method described by Baroni et al.^(20)^ Changes in dose distribution within CTV and OARs were assessed by considering DIBH variability related to all monitored sessions and to the single session with the highest difference in surface measurements compared to treatment plan (worst session). For each patient, the monitored session with the maximum 3D average distance between the planning and treatment positions of all passive markers was selected as the worst session. The treatment position was estimated by mediating passive marker coordinates over all DIBHs associated with the delivery of the treatment fields in each single session. The planning position was computed by manually segmenting in the DIBH CT images the radiopaque markers placed on the corresponding skin landmarks. Marker segmentation in CT volumes was performed through the medical image viewer VV,^(21)^ which allows a submillimeter resolution for image visualization and manual selection.

The rigid transform minimizing the Euclidean distance between the planning and treatment positions of all passive markers was estimated for each patient, considering the treatment positions both associated with the worst session and mediated over all monitored sessions. The obtained rotations and translations were applied to the original DIBH CT dataset and related structures. A replanning of the radiation treatment with identical beam and points of interest (isocenter and prescription points) was performed on the transformed CT volumes, obtaining the new dose‐volume histograms for CTV and OARs.

## III. RESULTS

The average total number of DIBHs acquired per patient was 35, ranging from 29 to 43. The length of the DIBHs was about 5 sec for SSD control and portal acquisition, whereas DIBHs performed during treatment delivery lasted 18‐20 sec or 11‐14 sec for patients treated with two or four tangential fields, respectively. The median (25th‐75th percentile) error in the 3D reconstruction of passive markers with the optoelectronic localizer, estimated during the daily calibration on the grid phantom, was 0.93 mm (0.63‐1.33 mm). The passive marker free‐breathing positions were stable in all patients, obtaining a free‐breathing stability (Eq. (6)) mediated over all passive markers lower than 0.7 mm for each spatial coordinate, with a median value across all patients of 0.3 mm for LL direction and 0.4 mm for AP and CC directions.

**Figure 3:**
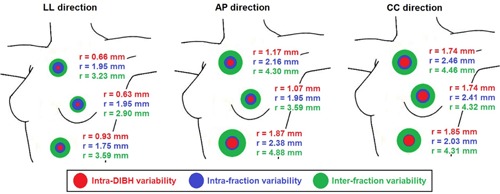
DIBH variability in passive marker positions for breast, thoracic, and abdominal regions. The radius (r) of the red circles represents the median value of the intra‐DIBH stability mediated over all DIBHs performed by each patient. The blue circles correspond to the median value of the intrafraction reproducibility over all monitored sessions of each patient, whereas the green circles indicate the median interfraction reproducibility.

Figure 3 reports the intra‐DIBH, intrafraction, and interfraction variability indices mediated over all patients along the three spatial directions, grouped by passive markers located on left‐breast, thorax, and abdomen (Fig 1(b)). The intra‐DIBH variability was lower than the other indices, with median values less than 1.9 mm along each direction. For the three anatomical regions, the ratio between intra‐ and interfraction errors was about 1:2, with the breast area showing variability closer to the thoracic region. The variability of the abdominal region was higher in AP direction, with median intra‐ and interfraction errors of 2.4 and 4.9 mm, respectively. The thoracic area, including the breast, showed an increased variability along CC direction, with median intra‐ and interfraction errors of 2.5 and 4.5 mm, respectively.

**Figure 4:**
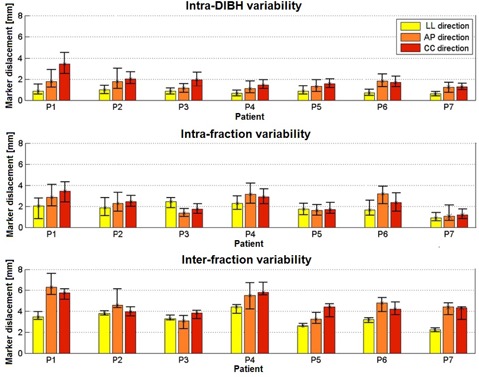
Intra‐DIBH, intrafraction, and interfraction DIBH variability (median±quartiles) along LL, AP, and CC directions mediated over all passive markers for each patient.

**Figure 5:**
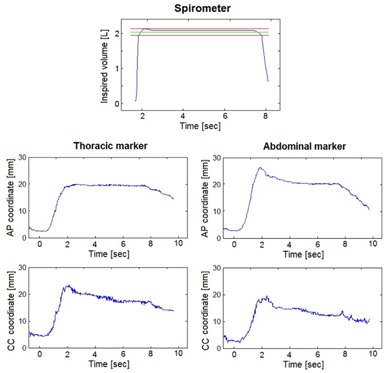
AP and CC motion of a thoracic and an abdominal passive marker during a DIBH performed by patient P1 at a constant inspired volume.

The variability indices mediated over all passive markers are depicted in Fig. 4 for each patient. The highest intra‐DIBH stability was found along LL direction, with median values ranging between 0.6‐1 mm. Along AP and CC directions, the intra‐DIBH variability in passive marker positions was less than 2 mm for all patients except patient P1, who had a median value of 3.5 mm along CC direction. An exemplificative DIBH performed by patient P1 is represented in Fig. 5, depicting variations of AP and CC coordinates of passive markers despite a constant level of inspired volume. The intrafraction reproducibility range across all patients was 0.9‐2.5 mm along LL direction, 1.1‐3.2 mm along AP direction, and 1.2‐3.4 mm along CC direction. The interfraction reproducibility range was 2.2‐4.4 mm, 3.1‐6.3 mm, and 3.8‐5.8 mm for LL, AP, and CC coordinates, respectively (Fig. 4).

The relationship between the measured surface displacements and the variability of the inspired volume within the tolerance band was also investigated. Linear correlation analysis between the intra‐DIBH positions of passive markers and the mean volumes inspired at corresponding DIBHs revealed that the two variables were statistically uncorrelated. Pearson coefficients computed for each single passive marker across all monitored sessions did not exceed 0.5, whereas Pearson values mediated over all passive markers were less than 0.4 for CC direction and 0.3 for AP and LL directions in all patients.

The 3D distances between the passive marker planning and treatment positions considering all monitored sessions ranged from 5.3 to 8.0 mm, with a mean value of 6.8 mm. The range of the 3D distances related to the worst session was 6.8‐14.0 mm, with a mean value of 9.5 mm. Table 1 reports for each patient the rotations and translations of the rigid transform computed between the passive marker planning and treatment positions associated with all monitored sessions. For four out of seven patients, the highest translations were found along CC axis, with a range between −6.3 and 5.4 mm. The remaining three patients showed higher translations in AP direction, ranging from −4.1 to 5.6 mm. The LL translation was less than 1.7 mm in all patients. The maximum absolute values of the measured rotations around LL, AP, and CC axes were 1.7°, 1.1°, and 2.1°, respectively.

The residual displacements, estimated as the 3D distance between the passive marker planning positions and the corresponding treatment positions after applying the rotations and translations of the rigid transform associated with all monitored sessions, are listed in Table 1 for each patient. The residual displacements mediated over all passive markers ranged between 1.9‐3.3 mm across all patients. Table 1 also includes the group mean, systematic, and random errors^(22)^ of the rotations and translations estimated for each spatial direction. These errors were obtained from the mean and standard deviation of the rotations and translations estimated for each patient between the passive marker planning positions and the corresponding intra‐DIBH positions associated with each treatment field delivery in all monitored sessions.

Table 2 lists for each patient the changes in CTV and OAR doses related to the estimated rotations and translations. We did not find any significant difference between dose variations obtained by considering all monitored sessions or the single worst session. As depicted in Table 2, in both cases the dosimetric indices averaged over all patients revealed a slight decrease in CTV coverage and OAR sparing compared to the original planning condition. $\text{CTV}\;D_{95\%}$ decreased by a maximum of 2.1% across all patients. Variations of ipsilateral lung $V_{20\text{Gy}}$ and contralateral breast $D_{1\text{cc}}$ did not exceed 2.5% and 1.8%, respectively. Changes in heart $V_{5\text{Gy}}$ were less than 1.2% for all patients except patient P4, who had a variation of +3%. For each dosimetric index, the measured differences met within 1% the prescribed dose constraints, except for CTV coverage in patient $P4\;(D_{95\%}=93.9)$, who showed, however, the same dosimetric condition also in the original treatment plan (Table 2).

**Table 1:** Rotations and translations between the treatment and planning positions of all passive markers. The median value±interquartile range (difference between 75 th and 25 th percentiles) of the residual displacements is listed for each patient. Group mean (M), systematic (Σ) and random (σ) errors for the rotations and translations estimated over all patients are also reported

	*Translation (mm)*			*Rotation (°)*			*Residual*
*Patient*	*LL Axis*	*AP Axis*	*CC Axis*	*LL Axis*	*AP Axis*	*CC Axis*	*Displacements (mm)*
P1	0.1	−4.1	−0.9	1.7	−1.1	−0.6	2.2±1.5
P2	0.4	−0.3	−4.4	−1.1	−0.4	0.4	2.2±1.0
P3	1.5	4.0	−5.1	−1.0	0.8	2.1	3.3±1.1
P4	0.9	−2.5	5.4	−1.5	−0.8	0.1	2.7±1.0
P5	1.7	5.6	−4.9	−1.6	0.1	1.3	1.9±2.3
P6	1.5	1.5	1.2	−1.4	−0.2	0.5	2.0±1.1
P7	0.6	1.3	−6.3	−0.6	0.3	−0.9	2.4±1.1
M	0.8	0.9	−2.2	−0.8	−0.4	0.4	
Σ	0.7	3.6	3.8	1.2	0.7	1.0	
σ	1.6	2.1	3.6	0.7	0.6	0.6	

**Table 2:** Dosimetric indices for CTV coverage and OAR sparing in the original treatment planning condition (C1), and after applying the rotations and translations derived from DIBH variability related to all monitored sessions (C2), and to the worst session (C3)

	$\text{CTV}\;D_{95\%}(\%)$			*Heart* $V_{5Gy}(\%)$			*Contralateral Breast* $D_{1cc}(\%)$			*Ipsilateral Lung* $V_{20Gy}(\%)$		
*Patient*	*C1*	*C2*	*C3*	*C1*	*C2*	*C3*	*C1*	*C2*	*C3*	*C1*	*C2*	*C3*
P1	96.0	95.9	96.0	0.9	1.0	0.9	6.2	3.0	6.9	10.6	12.0	13.1
P2	95.5	95.2	95.9	1.5	2.6	2.3	5.3	5.8	5.4	13.5	14.3	13.7
P3	95.3	94.5	95.3	0.1	0.8	1.3	4.0	5.3	5.3	9.2	6.5	7.2
P4	93.9	93.9	94.3	0.8	3.8	3.7	4.8	5.3	5.1	11.2	12.8	12.8
P5	98.2	96.1	96.5	0.4	0.8	1.4	3.8	4.1	4.3	10.0	8.1	9.5
P6	96.3	94.5	96.1	0.7	0.1	1.5	2.0	2.5	2.3	10.0	5.9	9.8
P7	97.7	95.8	96.5	1.6	1.9	0.6	5.8	7.6	7.1	9.3	8.1	7.1
Mean	96.1	95.1	95.8	0.9	1.6	1.7	4.6	4.8	5.2	10.5	9.7	10.5
Std	1.5	0.8	0.8	0.5	1.3	1.0	1.4	1.7	1.6	1.5	3.3	2.8

## IV. DISCUSSION

Stability and reproducibility of repeated DIBHs under spirometer‐based control were quantitatively evaluated on seven left‐breast cancer patients, assessing the variability in the position of multiple passive markers placed on the patients’ surface and acquired by means of IR optical tracking. Our results showed that in some cases the combined effect of intra‐ and inter‐DIBH variability during DIBHs performed at planned inspired volumes (± 0.1 liters) can lead to displacements of the external surface up to 6.3 mm along a single spatial direction. We are aware that the interpretation of the reported results may be influenced by a number of measurement uncertainties, including variability in passive marker repositioning on skin landmarks,^(23)^ limited spatial resolution of the couch shifts applied for setup corrections, and inaccuracies in radiopaque marker CT segmentation and passive marker optical localization. However, the applied methodological solutions (for example the subtraction of the free‐breathing baseline to reduce passive marker mispositioning and residual setup errors) allows limiting the size of these uncertainties, thus increasing the reliability of the reported results in terms of DIBH variability associated with spirometer‐based monitoring.

The reproducibility in the position of different anatomical structures (breast, nodal targets, heart, and arteries) under spirometric guidance has already been studied at various breath hold states by means of repeated CT scans and 2D portal images.^3^, ^24^, ^25^, ^26^ The innovative aspect of our approach is the use of noninvasive optical tracking for 3D surface monitoring, which permits a continuous and long‐term observation of DIBH reproducibility. The proposed method allows for the assessment of inter‐ and intra‐DIBH variability, as well as of setup uncertainties along all three spatial directions. These factors may explain the worse picture for DIBH reproducibility obtained in the present study with respect to the referenced works,^3^, ^24^, ^25^, ^26^ which were limited to 2D portal images or up to three CT images per patient.

The lack of correlation found between passive marker positions and inspired volumes suggests the estimated surface displacements do not depend on the variability of the inspiratory level reached within the tolerated range. For example, patient P3 exhibited surface displacements greater than 3 mm in each direction, but showed an interfraction variability of the mean inspired volume of 0.07 liters, which is significantly lower than the tolerance of 0.2 liters. Therefore, the reduction of the volumetric tolerance is not expected to provide any improvement in intra‐ and interfraction surface reproducibility. As shown in Fig. 5, even during a single DIBH, a constant inspired volume can be associated with surface displacements of up to 4 mm due to shoulder lowering and abdominal relaxation. This reinforces our finding that volume invariance during single or repeated DIBHs is not a reliable index of the geometric stability and reproducibility of patients’ thoraco‐abdominal surface.

The observed intra‐ and interfraction surface variability can be associated with the complexity of the respiration processes, especially during forced inspiration, involving the composed movements of the abdomen and thoracic rib cage.^(27)^ The same level of inspired volume can be reached with different combinations of thoracic elevation in CC direction and abdominal filling in AP direction. In the examined patient population, an inverse relationship between the AP coordinate of the abdominal passive markers and the CC coordinate of the thoracic passive markers was observed, with upper abdomen displacements during DIBHs being accompanied by caudal shifts of the thorax.

The performed dosimetric evaluation (Table 2) revealed that, in all patients, the measured surface displacements did not lead to significant changes in CTV coverage and OAR sparing. The prescribed dose constraints were met considering DIBH variability, both mediated over all treatment sessions and related to the single session with the highest surface differences compared to planning conditions. The margin of uncertainty for the interpretation of the obtained dosimetric alterations is given by the 3D residual displacements after correcting passive marker positions for the estimated rotations and translations (Table 1). The measured residual displacements are similar to the results of Baroni et al.,^(20)^ thus representing an acceptable range of confidence.

## IV. CONCLUSIONS

This work demonstrates that spirometric control for DIBH left‐breast radiotherapy treatments does not always guarantee a stable and reproducible position of the external breast surface. The CTV‐PTV clinical margins of 15 mm applied in the present study were adequate to cope with the measured DIBH variability under spirometer‐based guidance. However, when reduced margins and setup uncertainties are required for improving normal tissue sparing and decreasing cardiac and pulmonary toxicity in DIBH radiotherapy, the reported results suggest the need for more robust DIBH monitoring methods. For example, spirometric techniques can be integrated with the IR localization of multiple passive markers placed in proximity to the target volume.^(28)^ Optical surface imaging systems can also be applied to increase surface position reproducibility during repeated DIBHs,^12^, ^13^ especially if combined with deformable registration algorithms for the extraction of multidimensional breathing signals from markerless surface acquisitions.^(29)^
